# Diagnosing high-grade pancreatic intraepithelial neoplasia in surgically altered anatomy using pancreatic juice cytology

**DOI:** 10.1016/j.vgie.2025.02.006

**Published:** 2025-02-21

**Authors:** Soma Fukuda, Susumu Hijioka, Kohei Okamoto, Shin Yagi, Mark Chatto, Yutaka Saito, Takuji Okusaka

**Affiliations:** 1 Department of Hepatobiliary and Pancreatic Oncology, National Cancer Center Hospital, Tokyo, Japan; 2 Department of Medicine, Makati Medical Center, Manila, Philippines; 3 Endoscopy Division, National Cancer Center Hospital, Tokyo, Japan

## Abstract

**Background and Aims:**

Diagnosing early-stage pancreatic ductal adenocarcinoma, particularly high-grade pancreatic intraepithelial neoplasia (HG-PanIN), remains challenging. Serial pancreatic juice aspiration cytologic examination (SPACE) using an endoscopic nasopancreatic drainage tube has demonstrated high diagnostic accuracy, but its application in surgically altered anatomy is technically demanding. We present a case in which balloon enteroscopy-assisted SPACE led to the diagnosis of HG-PanIN and successful resection.

**Methods:**

A 70-year-old man with a history of distal gastrectomy and Roux-en-Y reconstruction for gastric cancer underwent follow-up imaging, which revealed localized main pancreatic duct (MPD) stricture and parenchymal atrophy in the pancreatic tail. EUS identified a faint hypoechoic area around the stricture, but no distinct mass. EUS-guided tissue acquisition was inconclusive. Double-balloon enteroscopy-assisted endoscopic retrograde pancreatography was performed, revealing MPD stricture and distal dilation. A 5F endoscopic nasopancreatic drainage tube was placed across the stricture, and SPACE was conducted.

**Results:**

Twelve pancreatic juice cytology samples were aspirated every 2 to 3 hours over 3 days, each exceeding 1 mL. One sample (10th) was classified as Class IV, “suspicious for adenocarcinoma,” with cytology revealing nuclear enlargement and atypia. The patient was diagnosed preoperatively with pancreatic cancer (TisN0M0 stage 0) and underwent distal pancreatectomy without neoadjuvant chemotherapy. Pathology confirmed HG-PanIN of the MPD. The patient had no postoperative adverse events and remained recurrence-free at the 9-month follow-up.

**Conclusions:**

This case highlights the effectiveness of balloon enteroscopy-assisted SPACE in diagnosing HG-PanIN in surgically altered anatomy. However, given the relatively high risk of pancreatitis, SPACE should be reserved for patients with imaging or clinical findings suggestive of malignancy. By overcoming technical obstacles, this method offers a promising diagnostic strategy for early-stage pancreatic ductal adenocarcinoma in surgically altered anatomy.

Diagnosing early-stage pancreatic ductal adenocarcinoma (PDAC), especially high-grade pancreatic intraepithelial neoplasia (HG-PanIN), remains challenging.^1^^,^^2^ HG-PanIN poses a high risk of progressing to invasive carcinoma and warrants surgical treatment.^3^ Few reports of HG-PanIN diagnosed through endoscopic ultrasound-guided tissue acquisition (EUS-TA) exist; moreover, obtaining cancer cells from the pancreatic duct is difficult.^4^^,^^5^ Serial pancreatic juice aspiration cytologic examination (SPACE), using an endoscopic nasopancreatic drainage (ENPD) tube, demonstrates diagnostic sensitivity of 62.4% to 82.4% for early-stage PDAC.^6^^,^^7^ However, SPACE in surgically altered anatomy is technically demanding, with limited reports published.^8^ Herein, we present a case in which balloon enteroscopy-assisted SPACE led to a diagnosis of HG-PanIN and its successful resection. This study was approved by our institutional review board (approval no. 2018-149).

A 70-year-old man underwent distal gastrectomy with Roux-en-Y reconstruction 17 years earlier for gastric cancer. Follow-up computed tomography, despite the patient reporting no symptoms, showed localized main pancreatic duct (MPD) stricture and parenchymal atrophy in the pancreatic tail, and magnetic resonance cholangiopancreatography confirmed the MPD stricture and distal dilatation without any detectable mass (Fig. 1). EUS identified a faint hypoechoic area around the MPD stricture in the pancreatic tail but no distinct mass. Because malignancy could not be excluded, EUS-TA was attempted. The hypoechoic area was punctured transgastrically 4 times using a 22-gauge Franseen needle (SonoTip TopGain; Medico's Hirata, Osaka, Japan); however, the results were inconclusive (Fig. 2).

**Figure 1:**
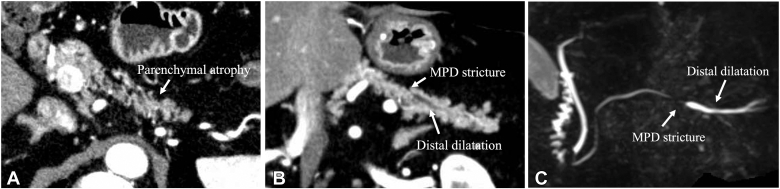
Contrast-enhanced CT and MRCP findings. **A** and **B,** Axial and coronal CT images showing the MPD stricture and parenchymal atrophy in the pancreatic tail. **C,** MRCP revealing the MPD stricture and distal dilatation. *CT*, Computed tomography; *MPD*, main pancreatic duct; *MRCP,* magnetic resonance cholangiopancreatography.

**Figure 2:**
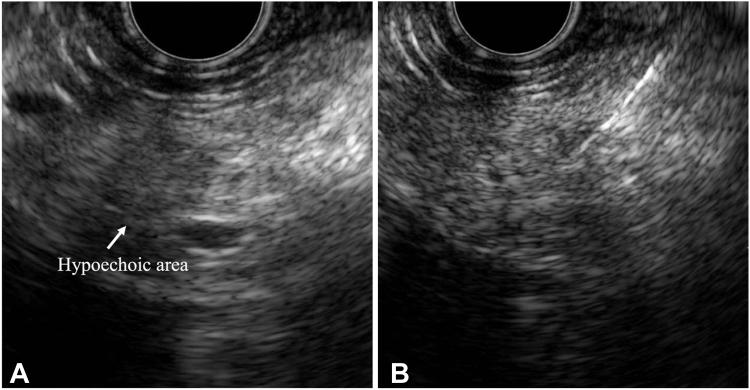
EUS findings. **A,** EUS showing a faint hypoechoic area around the main pancreatic duct stricture, with no distinct mass. **B,** EUS-guided tissue acquisition was performed, but results were inconclusive.

We proceeded with double-balloon enteroscopy-assisted endoscopic retrograde pancreatography (Fig. 3). A short-type double-ballon enteroscopy (EI-580BT; Fujifilm, Tokyo, Japan) was used. Scope insertion was challenging because of postsurgical adhesions, requiring careful maneuvering to reach the major papilla. Although catheter and duct misalignment made pancreatic duct cannulation difficult, a papillotome (CleverCut 3V; Olympus, Tokyo, Japan) enabled biliary cannulation. A 0.025-inch guidewire (J-WIRE Prologue ST; J-MIT, Shiga, Japan) was inserted into the bile duct, and pancreatic duct cannulation was eventually achieved with papillotome and biliary guidewire assistance. Pancreatography confirmed MPD stricture and distal dilatation (Fig. 4, Video 1, available online at www.videogie.org). A 5F ENPD tube (Geenen; Cook Medical, Bloomington, Ind, USA) was placed across the stricture without performing pancreatic sphincterotomy, and the procedure was completed uneventfully.

**Figure 3:**
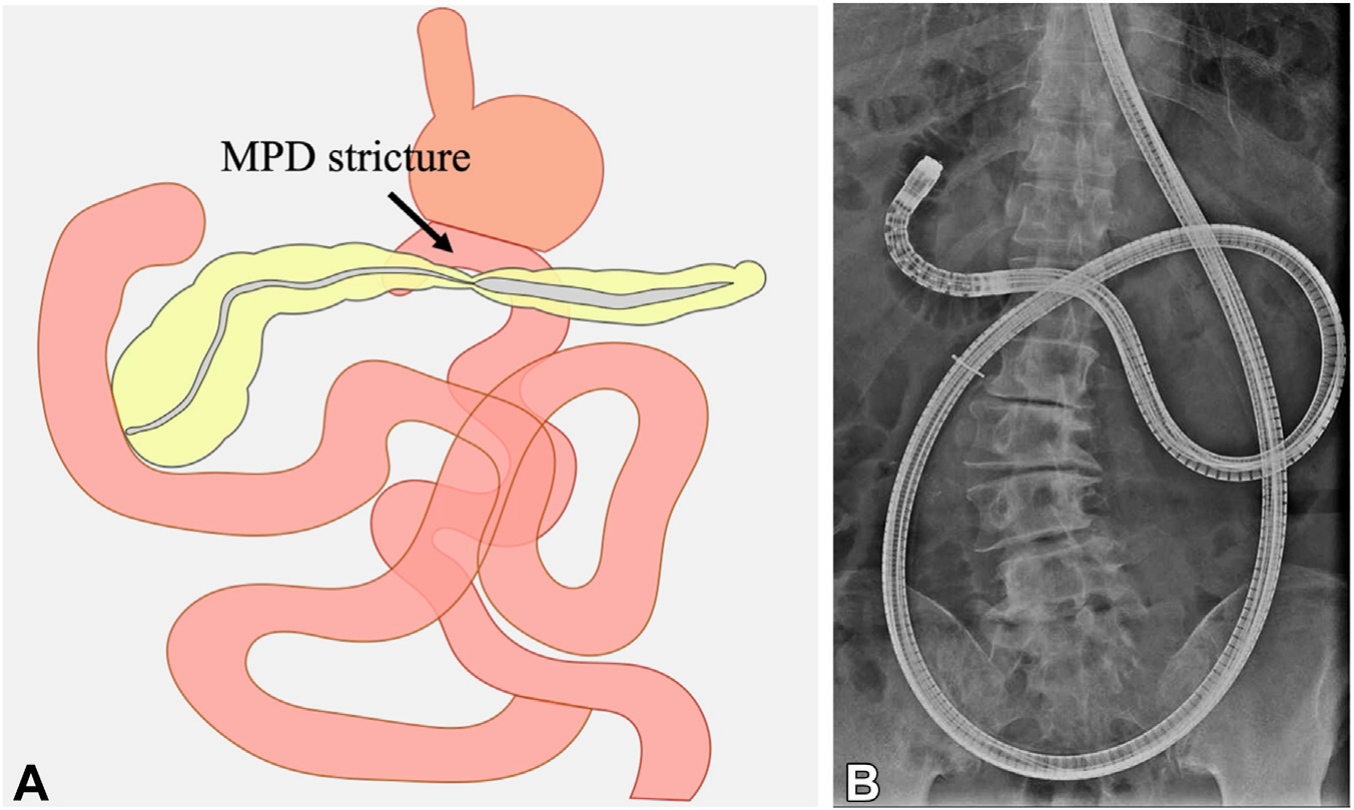
Plan for double-balloon enteroscopy-assisted endoscopic retrograde pancreatography. **A,** Schema of the anatomical layout in the present case. **B,** Fluoroscopic image showing a double-balloon enteroscope inserted into the duodenum. *MPD*, Main pancreatic duct.

**Figure 4:**
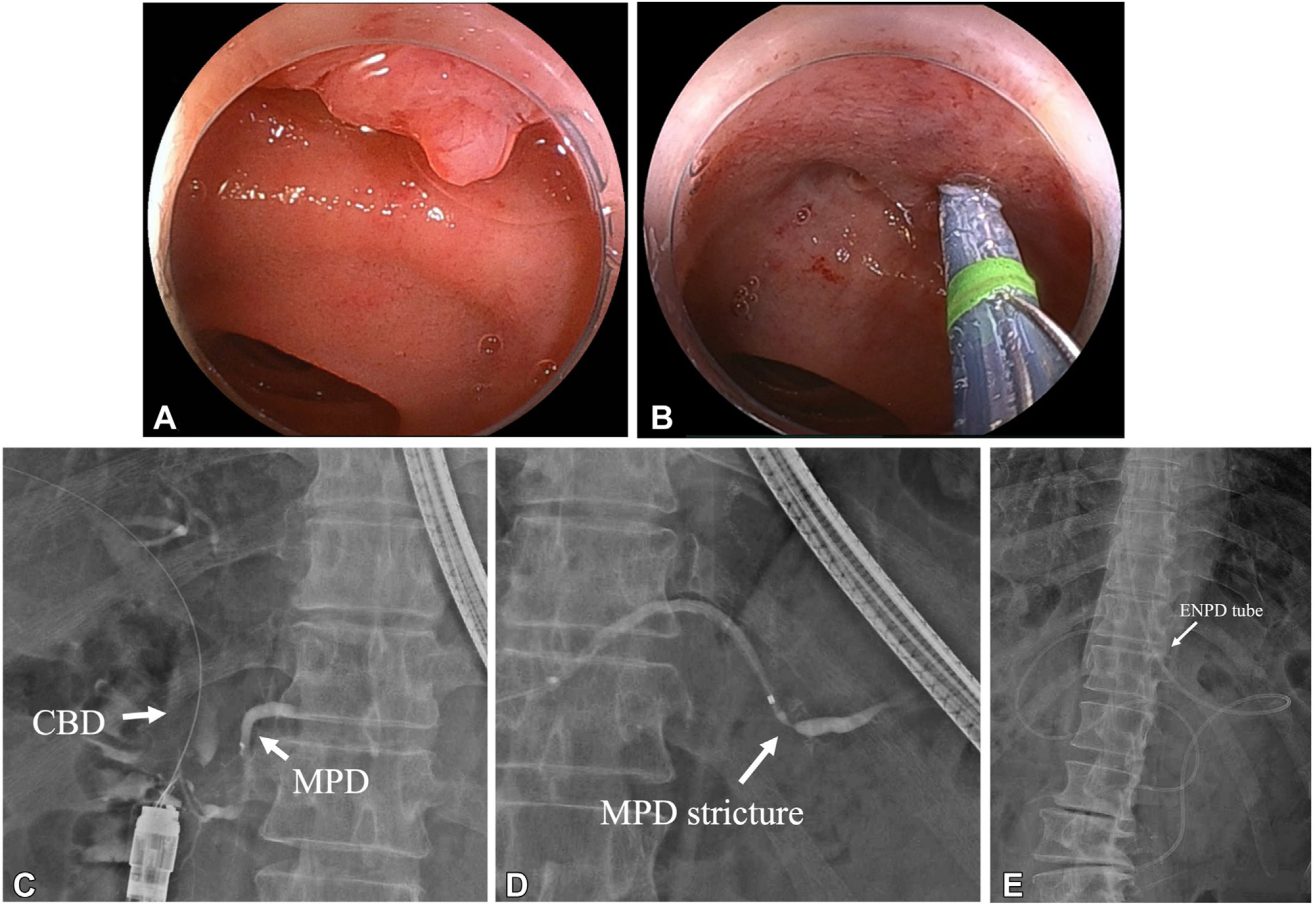
Endoscopic and fluoroscopic images of double-balloon enteroscopy-assisted endoscopic retrograde pancreatography. **A,** Endoscopic image of the major papilla. **B,** Bile duct cannulation using a papillotome. **C,** Pancreatic duct cannulation achieved with biliary guidewire assistance. **D,** Pancreatography showing the MPD stricture and distal MPD dilatation in the pancreatic tail. **E,** Fluoroscopic image after placement of a 5F ENPD tube. *CBD*, Common bile duct; *ENPD*, endoscopic nasopancreatic drainage; *MPD*, main pancreatic duct.

SPACE was performed during hospitalization, with 12 pancreatic juice cytology samples aspirated via the ENPD tube every 2 to 3 hours over 3 days, each exceeding 1 mL, and the tube removed bedside.^6^ No postprocedure pancreatitis occurred. One of the 12 samples was classified as class IV, “suspicious for adenocarcinoma,” with cytology revealing cells with nuclear enlargement and atypia, leading to a preoperative diagnosis of pancreatic cancer TisN0M0 stage 0 (Fig. 5). Without neoadjuvant chemotherapy, the patient underwent distal pancreatectomy 2 months later. Pathology confirmed HG-PanIN of the MPD in a single section, corresponding to the MPD stricture (Fig. 6). The patient had no postoperative adverse events. At the 9-month follow-up, no recurrence was noted, and the patient remains in good health.

**Figure 5:**
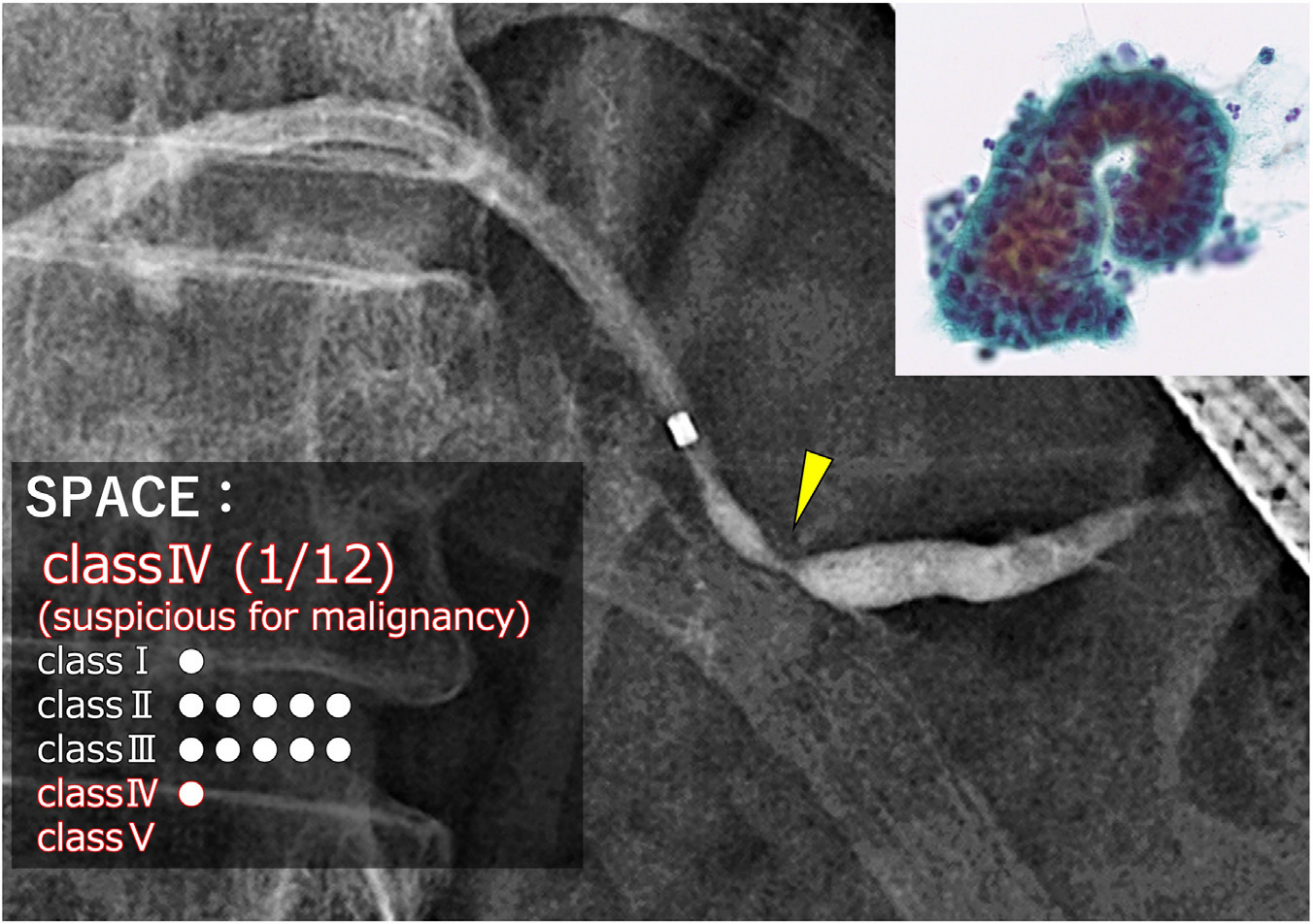
Cytologic evaluation of a SPACE sample, classified as Class IV, “suspicious for adenocarcinoma.” *SPACE*, Serial pancreatic juice aspiration cytologic examination.

**Figure 6:**
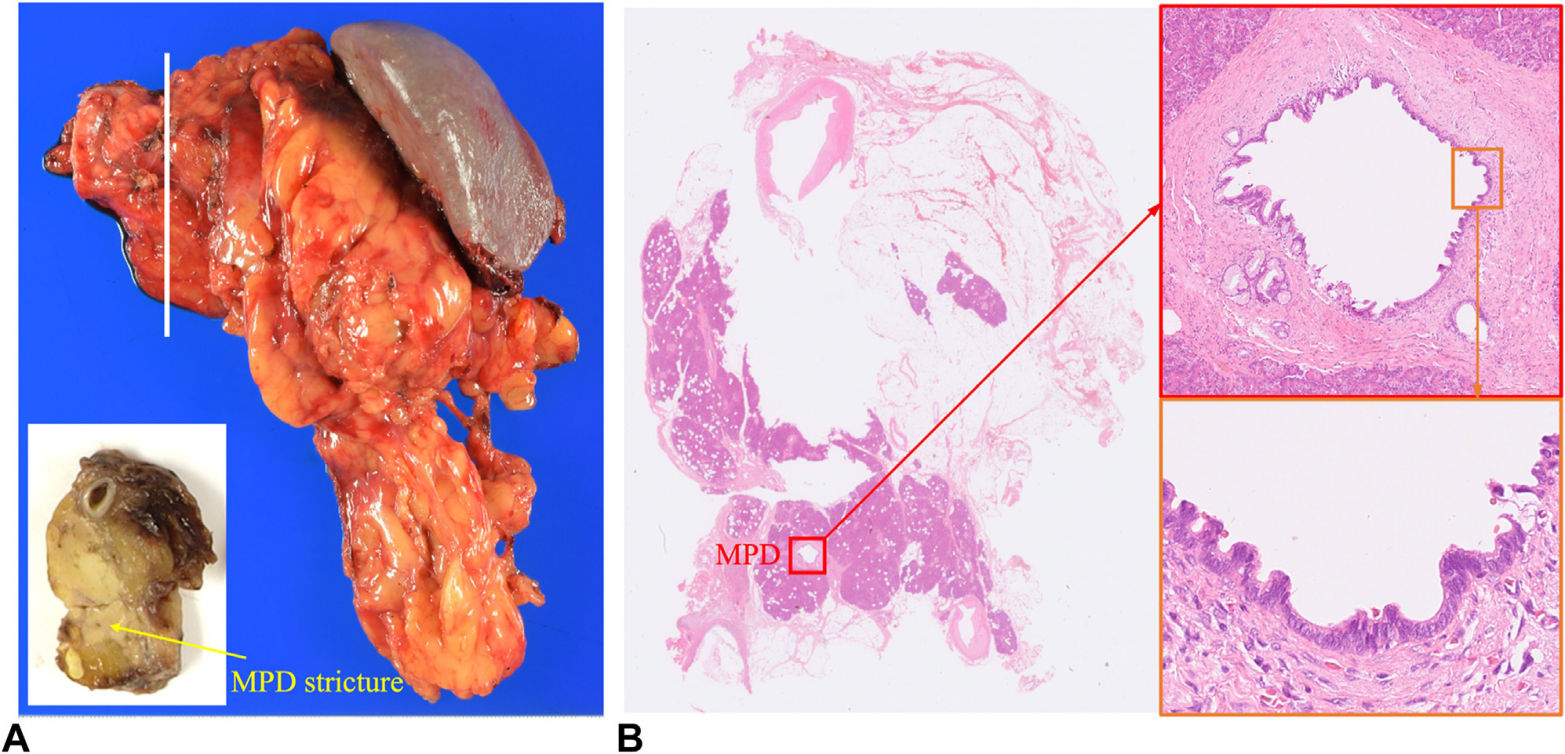
Pathological findings of the surgical specimen. **A,** Surgical specimen after distal pancreatectomy, including a section of the MPD stricture site. **B,** Histopathologic examination confirming high-grade pancreatic intraepithelial neoplasia of the MPD, corresponding to the stricture site shown in (**A**). *MPD*, Main pancreatic duct.

In imaging-based diagnosis of HG-PanIN, key indirect findings include MPD stenosis and localized pancreatic atrophy surrounding a hypoechoic area on EUS.^9^ A multicenter study in Japan showed SPACE has superior sensitivity for diagnosing HG-PanIN compared with EUS-TA (72.2% vs 16.7%).^10^ Given the relatively high pancreatitis risk of 0% to 16%,^7^ SPACE should be reserved for patients with imaging or clinical findings suggestive of malignancy.

In this case, EUS revealed indirect findings suggestive of HG-PanIN, such as localized pancreatic atrophy and MPD stenosis. Given the challenges of performing SPACE in surgically altered anatomy, EUS-TA was initially attempted but failed to provide a definitive diagnosis. Ultimately, balloon enteroscopy-assisted SPACE confirmed HG-PanIN diagnosis. By overcoming technical obstacles, this method offers a promising diagnostic strategy for early-stage PDAC in surgically altered anatomy, potentially improving outcomes.

## Patient Consent

The patient in this article has given written informed consent to publication of the case details.

## Disclosure

This work was supported in part by The National Cancer Center Research and Development Fund 2022-A-16. The authors have no conflicts of interest to disclose.

## Supplementary data

Video 1 A case of high-grade pancreatic intraepithelial neoplasia (HG-PanIN) diagnosed in surgically altered anatomy using balloon enteroscopy-assisted serial pancreatic juice aspiration cytologic examination (SPACE)

## References

[1] Kawasaki Y., Hijioka S., Nagashio Y. (2024). Diagnostic performance of EUS-guided tissue acquisition for solid pancreatic lesions ≤10 mm. Endosc Ultrasound.

[2] Sagami R., Nakahodo J., Minami R. (2024). True diagnostic ability of EUS-guided fine-needle aspiration/biopsy sampling for small pancreatic lesions ≤10 mm and salvage diagnosis by pancreatic juice cytology: a multicenter study. Gastrointest Endosc.

[3] Basturk O., Hong S.M., Wood L.D. (2015). A revised classification system and recommendations from the baltimore consensus meeting for neoplastic precursor lesions in the pancreas. Am J Surg Pathol.

[4] Sakamoto H., Kitano M., Dote K. (2008). In situ carcinoma of pancreas diagnosed by EUS−FNA. Endoscopy.

[5] Kitamura H., Hijioka S., Nagashio Y. (2022). A case of high grade pancreatic intraepithelial neoplasia diagnosed by endoscopic ultrasound-guided fine needle aspiration. Endoscopy.

[6] Iiboshi T., Hanada K., Fukuda T. (2012). Value of cytodiagnosis using endoscopic nasopancreatic drainage for early diagnosis of pancreatic cancer. Pancreas.

[7] Hanada K., Shimizu A., Kurihara K. (2022). Endoscopic approach in the diagnosis of high-grade pancreatic intraepithelial neoplasia. Dig Endosc.

[8] Mie T., Sasaki T., Sasahira N. (2023). Serial pancreatic juice aspiration cytologic examination with balloon-assisted enteroscopy in surgically altered anatomy. Dig Endosc.

[9] Nakahodo J., Kikuyama M., Nojiri S. (2020). Focal parenchymal atrophy of pancreas: an important sign of underlying high-grade pancreatic intraepithelial neoplasia without invasive carcinoma, i.e., carcinoma in situ. Pancreatology.

[10] Kanno A., Masamune A., Hanada K. (2018). Multicenter study of early pancreatic cancer in Japan. Pancreatology.

